# An Observational Survey Study on the Use of Locoregional Anaesthesia in Non-Conventional Species: Current Practice and Potential Future Developments

**DOI:** 10.3390/ani12111448

**Published:** 2022-06-03

**Authors:** Nuria Quesada, Dario d’Ovidio, Matt Read, Paolo Monticelli, Chiara Adami

**Affiliations:** 1 Royal Veterinary College, Hawkshead Lane, Hatfield AL9 7TA, UK; nquesadavicent@rvc.ac.uk; 2 Independent Researcher, 80022 Arzano, Italy; dariodovidio@yahoo.it; 3 MedVet Columbus, Worthington, OH 43085, USA; matt.read@medvet.com; 4 Dick White Referrals, London Road, Cambridgeshire CB8 0UH, UK; paolo.monticelli@gmail.com; 5 Department of Veterinary Medicine, University of Cambridge, Madingley Road, Cambridge CB3 0ES, UK

**Keywords:** exotic pet, analgesia, local anaesthetic, locoregional anaesthesia, nerve block, zoo animal

## Abstract

**Simple Summary:**

Locoregional anaesthesia is a useful tool to improve perioperative analgesia, and its use continues to progressively increase in both small animal pets and human patients. However, whether and how locoregional anaesthesia is being used in non-conventional animal species is largely unknown. This study was designed to answer these questions, as well as to identify specific research areas on this topic that would be useful to increase the use of locoregional anaesthesia in non-domestic species. This study revealed that locoregional anaesthesia is still relatively underutilised in non-conventional animal species, although there is a tendency to utilise specific blocks routinely, especially in rabbits and rodents, which have become popular as pets. Reluctance to perform locoregional anaesthesia was reported to be due to a lack of species-specific information on effective and toxic doses of local anaesthetics and a paucity of blocks that are specifically developed for non-conventional species. It is concluded that novel studies that focus on species-specific, applied pharmacology of local anaesthetics and block techniques—particularly dental blocks for rabbits and rodents and blocks for the wings of birds—could promote more widespread use of locoregional anaesthetic techniques in non-conventional animal species.

**Abstract:**

The objectives of this study were to investigate the current attitudes of veterinarians towards the use of locoregional anaesthesia in non-conventional animal species and to identify areas for future useful research on this topic. A questionnaire was circulated online. A total of 417 veterinarians, including American and European specialists/specialists-in-training in both zoological medicine and anaesthesia/analgesia (ACZM/ECZM and ACVAA/ECVAA), participated in the study. Fifty-nine percent of respondents performed locoregional anaesthesia in rabbits, with intratesticular injections and local infiltration being the most commonly-reported techniques. ACZM/ECZM specialists reportedly performed dental blocks in rabbits more frequently than ACVAA/ECVAA specialists (*p* = 0.030). Forty percent of respondents performed locoregional anaesthesia in rodents, with intratesticular injections, topical/splash blocks and local infiltration being the most commonly reported techniques. The proportions of respondents who reportedly used locoregional anaesthesia in ferrets, birds and reptiles were 37.9%, 34.5% and 31.2%, respectively. The use of Tuohy (*p* < 0.001) and spinal needles (*p* < 0.001), as well as of ultrasonography (*p* = 0.009) and nerve-stimulators (*p* < 0.001), was more common among ACVAA/ECVAA compared to ACZM/ECZM specialists. Major topic areas for future research were identified as dental block techniques for rabbits and rodents and blocks for the wings of birds.

## 1. Introduction

Locoregional anaesthesia has been used for decades in animals, owing to its beneficial effects on patient outcomes [1]. It has been proven to decrease systemic analgesic requirements and postoperative opioid consumption [2,3]. Moreover, in both dogs and human patients, the use of locoregional anaesthetic techniques in the perioperative period has been found to reduce the cost and length of hospitalisation [4,5].

Over the past decade, novel approaches to performing nerve blocks, including the use of nerve stimulation and ultrasound guidance, have been developed for use in both people and animals [6,7,8,9,10]. However, the literature on the use of locoregional anaesthesia in non-conventional animal species is scarce, despite these species becoming increasingly popular as pets [11].

A previous survey study on the use of anaesthesia and analgesia in reptiles reported that only 32.7% of respondents used locoregional anaesthesia in this taxonomic group [12]. Surprisingly, survey studies that investigated analgesia in rabbits and guinea pigs did not include locoregional anaesthesia among the options for pain management [13,14]. Moreover, one study reported that in laboratory rabbits, locoregional anaesthesia was used more frequently in the late 1990s compared to the period between 2005 and 2007 [15]. Overall, these findings seem to suggest that, despite locoregional anaesthesia playing a crucial role in modern pain management for domestic small and large animals, it may still be underused in non-conventional animal species.

The aims of this study were to:

- Investigate the current attitudes of veterinarians who commonly perform anaesthesia in non-conventional animal species towards using locoregional anaesthesia, and
- Identify areas for potentially useful future research, based on the challenges faced by the respondents when performing locoregional anaesthesia in non-conventional animal species and the specific areas for which they perceive that there are knowledge gaps.

We hypothesised that most veterinarians do not use locoregional anaesthesia routinely in non-conventional animal species and that this is the result of the low number of techniques specifically developed for non-conventional species and the limited information that is currently available regarding species-specific applied pharmacology of local anaesthetic drugs.

## 2. Materials and Methods

### 2.1. Study Design

This observational study was designed to capture information using an online survey tool/questionnaire developed by the authors (SurveyMonkey; Momentive Inc., San Mateo, CA, USA). Both the questionnaire and the study were designed based on the Checklist for Reporting Results of Internet E-Surveys (CHERRIES) [16]. Ethical approval for the study was obtained from the Social Science Research Ethical Review Board of the Royal Veterinary College (RVC-SSRERB; license number: URN SR 2020-0263).

### 2.2. Advertisement and Recruitment of Participants

The target population of respondents for this study was veterinarians with a moderate-to-high degree of exposure to non-conventional animal species (and who had a high likelihood of performing anaesthesia on them), or veterinarians who performed anaesthesia at an advanced level who would also have frequent exposure to non-conventional animal species. A total of 236 veterinarians, including diplomates and residents in-training of the American and European Colleges of both Veterinary Anaesthesia and Analgesia (ACVAA and ECVAA) and Zoological Medicine (ACZM and ECZM), were contacted via email and invited to participate in the study. Additionally, a link to the survey was created and made available on the ECZM website, the social media platforms of both the ECZM and the ECVAA residents’ groups and through the ACVAA and ECVAA email lists.

Participation was entirely voluntary and no incentives were offered to potential participants. In order to prevent more than one set of responses from being entered by the same person, the survey was set to detect multiple entries being linked to the same email address.

### 2.3. Data Protection and Anonymity

Before commencing the survey, each participant was asked to provide permission for the use and publication of data, according to the General Data Protection Regulations (GDPR EU 2016/679). Clicking on the agreement checkbox on the introductory page of the survey was a formal requirement prior to accessing the questionnaire. In order to prevent unauthorised access to personal information and potentially sensitive data, the access to the data generated by the survey was password-protected. The answers to the questions were automatically delinked from the participants’ email addresses to ensure confidentiality. Additionally, the email addresses were not disclosed to the investigators to prevent potential identification of the participants.

### 2.4. Questionnaire

The questionnaire was composed of three sections with a total of 30 multiple choice questions. Ordinal scales such as the Likert scale were used to formulate the possible answers [17]. The templates used for the Likert scale included level of agreement, level of importance, level of reflectance of the participant’s belief and frequency [18].

In the first section, which included 11 questions, general information and demographic data were obtained, including country of practice, type of specialty board certification(s) (if attained), attitudes towards pain management and work experience with various animal species across seven different taxonomic groups—rabbits, ferrets, lizards, snakes, chelonians, birds and rodents (which included guinea pigs, chinchillas, rats, mice, hamsters and gerbils).

The second section was comprised of 12 questions that focused on the respondents’ current practice in relation to the use of locoregional techniques in the aforementioned taxonomic groups, the frequency with which they performed locoregional anaesthesia and the types of drugs and equipment that they used.

In the third section, which included seven questions, participants were asked about which aspects of locoregional anaesthesia in non-conventional species would, in their opinion, benefit from further development and research. Moreover, the reasons behind a potential reluctance to perform locoregional anaesthesia in non-conventional species were investigated.

### 2.5. Statistical Methods

The questionnaire was designed to generate quantitative data that could be assessed using non-parametric statistics. Qualitative data were limited to demographic variables and analysed where appropriate using descriptive statistics. The completion rate was calculated. Data distribution was analysed with the Shapiro-Wilk test. A Chi-squared test was used to investigate whether there were differences between ACVAA/ECVAA and ACZM/ECZM specialists (including both the diploma holders and the specialists-in- training) with respect to the frequency with which specific blocks were performed, technique, and the current reason for which specific areas would benefit from further development and research. Commercially available software (SigmaStat 4.0, Systat Software, San Jose, CA, USA, and IBM SPSS Statistics for Windows, Version 26.0, Armonk, NY, USA) were used. *p* < 0.05 were considered to be statistically significant.

## 3. Results

The overall completion rate of the survey was 72%, with not all respondents replying to all questions. Since not all questions were answered by all the participants, when numbers (*n*) are reported alongside proportions (%), the denominator indicates the total number of participants who answered the specific question.

The words “consistent use/consistently” are used in this study to define a frequency classified by the participants as being either “always/often” or “almost every time/every time”.

### 3.1. General Information

The survey remained open from December 2020 to April 2021. During this time, a total of 417 veterinarians participated in the survey and took an average time of 11 min and 42 s to complete the questionnaire. The majority of the participants worked in Europe (65.2%; *n* = 272/417), followed by North America (29.0%; *n* = 121/417), Australasia (3.1%; *n* = 13/417), South America (1.2%; *n* = 5/417), Asia (1.0%; *n* = 4/417) and Africa (0.5%; *n* = 2/417).

It was found that 55.6% of respondents (*n* = 232/417) held at least one level of specialty board certification: 56.5% (*n* = 131/232) were ACVAA/ECVAA diplomates, 20.7% (*n* = 48/232) were ACZM/ECZM diplomates, 0.86% (*n* = 2/232) were diplomates of the American Board of Veterinary Practitioners (ABVP) and the remaining 22.0% (*n* = 51/232) held another (non-specified) type of board certification. Residents-in-training represented 14.4% (*n* = 60/417) of the total sample population, of which 93.3% (*n* = 56/60) were ACVAA or ECVAA residents, 5.0% (*n* = 3/60) were registered with either ACZM or ECZM training programs and the remaining 1.7% (*n* = 1/60) was an ABVP resident. The remaining 30.0% of participants (*n* = 125/417) were neither board-certified specialists nor specialists-in-training.

The scope of practice of respondents was varied, with 58.0% (*n* = 242/417) working in small animal practices that treated exotic species, 17.7% (*n* = 74/417) working in mixed-species practices, 7.0% (*n* = 29/417) working in zoos and 2.4% (*n* = 10/417) working with wildlife. The remaining 14.9% (*n* = 62/417) were classified as “other type of practice”.

**Figure 1:**
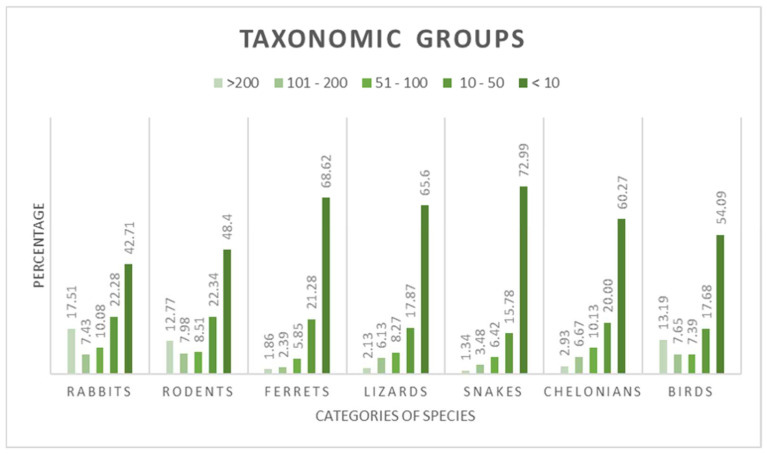
Relative exposure of the respondents (*n* = 417) to non-conventional animals based on their species/taxonomic group. The percentages on the *y*-axis indicate the proportions of veterinarians participating to the study who reportedly performed anaesthesia in each specific species/categories of species. <10, 10–50, 51–100, 101–200 and >200 are the number of animals, within each species/taxonomic group, anaesthetised per year by the respondents.

Rabbits, rodents and birds were the species most frequently anaesthetised, whereas ferrets and snakes were the least frequently anaesthetised (Figure 1).

The vast majority of the veterinarians who participated in the survey believed that pain is consciously perceived by rabbits (99.0%; *n* = 413/417), rodents (98.8%; *n* = 412/417), ferrets (98.8%; *n* = 412/417), lizards (96.9%; *n* = 404/417) and snakes (96.9%; *n* = 404/417). However, only 58.0% (*n* = 242/417) of respondents considered their knowledge on pain management to be adequate. Furthermore, 21.3% (*n* = 89/417) of respondents claimed that the majority of their clients (76%–100%) expressed concern about pain management in their non-conventional pets; however, 20.6% (*n* = 86/417) reported that less than 25% of their clients were concerned about it.

**Figure 2:**
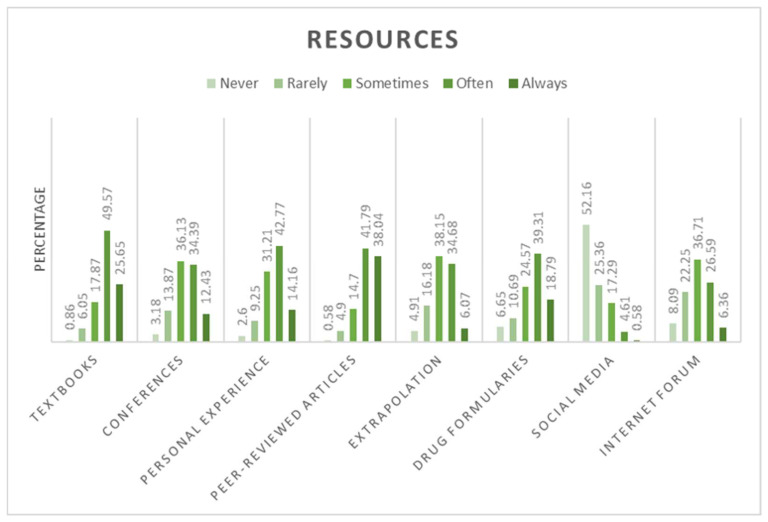
Resources used by the respondents (*n* = 417) to obtain information about analgesia and locoregional anaesthesia as it applies to non-conventional animal species. Extrapolation: extrapolation of techniques and drug doses from domestic animal species. The percentages on the *y*-axis indicate the proportions of veterinarians who reportedly used each specific resource on the *x*-axis. Never, rarely, sometimes, often and always indicate the frequency of use of the resources listed on the *x*-axis.

The sources that respondents consulted to obtain information on locoregional techniques in non-conventional species were original research and scientific peer-reviewed articles, textbooks, drug formularies, personal clinical experiences, conference proceedings, internet discussion and forums or webinars. However, participants also used their past clinical experience and extrapolated notions and dosages from domestic animal species (Figure 2).

### 3.2. Current Practice and Use of Locoregional Techniques

#### 3.2.1. Rabbits

It was found that 59.0% (*n* = 246/417) of respondents performed locoregional anaesthesia in rabbits. The most commonly performed techniques were intratesticular injections and local infiltration (65.5%; *n* = 161/246 and 52.4%; *n* = 129/246), respectively). Peripheral nerve blocks of the thoracic and pelvic limbs were the least performed, with 59.4% (*n* = 146/246) and 52.0% (*n* = 128/246) of the participants claiming that they used these techniques either rarely or never. Only 18.3% (*n* = 45/246) of respondents performed neuraxial anaesthesia in rabbits.

There was a statistically significant difference between the proportion of ACVAA/ECVAA and ACZM/EZCM specialists/specialists-in-training who consistently performed dental blocks in rabbits (20.2%, *n* = 28/72 vs. 51.9%, *n* = 14/27, respectively; *p* = 0.03).

#### 3.2.2. Rodents

Forty percent of the respondents (*n* = 167/417) performed locoregional anaesthesia in rodents. The techniques most commonly performed were intratesticular injections, topical/splash blocks and local infiltration (58.7%; *n* = 98/167, 56.3%; *n* = 94/167 and 53.3%; *n* = 89/167, respectively). Neuraxial anaesthesia was the least commonly performed technique, followed by blocks of the ear/ear canal and of both the thoracic and pelvic limbs, with 76.0% (*n* = 127/167), 70.1% (*n* = 117/167), 66.5% (*n* = 111/167) and 59.9% (*n* = 100/167) of respondents claiming that they rarely or never use these techniques.

There were statistically significant differences between the proportions of ACVAA/ECVAA and ACZM/EZCM specialists/specialists-in-training who consistently performed dental blocks (20.8%, *n* = 11/53 vs. 29.0%, *n* = 9/31, respectively; *p* = 0.038) and ear/ear canal blocks in rodents (6.8%, *n* = 4/59 vs. 12.9%, *n* = 4/31, respectively; *p* = 0.044).

#### 3.2.3. Ferrets

It was found that 37.9% of respondents (*n* = 158/417) performed locoregional anaesthesia in ferrets. The most popular techniques were local infiltration, topical/splash blocks and intratesticular injections (53.2%; *n* = 84/158, 48.1%; *n* = 76/158 and 41.8%; *n* = 66/158, respectively). Ear/ear canal blocks and neuraxial anaesthesia were the least commonly-used techniques, with 70.9% (*n* = 112/158) and 63.9% (*n*= 101/158) of respondents reporting that they rarely or never performed these techniques.

There were statistically significant differences between the proportions of ACVAA/ECVAA and ACZM/EZCM specialists/specialists-in-training who consistently performed intratesticular injections (39.7%, *n* = 25/63 vs. 9.7%, *n* = 3/31, respectively; *p* = 0.003) and neuraxial anaesthesia in ferrets (26.6%, *n* = 17/64 vs. 9.7%, *n* = 3/31, respectively; *p* = 0.017).

#### 3.2.4. Reptiles

Only 31.2% of respondents (*n* = 130/417) performed locoregional anaesthesia in reptiles. Topical splash blocks and local infiltration were the techniques most commonly performed (47.7%; *n* = 62/130 and 43.1%; *n* = 56/130, respectively). Dental, eye, intrathecal and front limb blocks were the least common techniques used, with 80.0% (*n* = 104/130), 74.6% (*n* = 97/130), 60.8% (*n* = 79/130) and 57.7% (*n* = 75/130) of respondents reporting that they rarely or never performed these techniques in reptiles.

#### 3.2.5. Birds

It was found that 34.5% of respondents (*n* = 144/417) performed locoregional anaesthesia in birds. The most commonly performed technique was local infiltration, with 29.9% of respondents (*n* = 43/144) reporting its consistent use in birds. The least performed techniques were those of the eye/globe and intrathecal anaesthesia, with 73.4% (*n* = 110/144) and 90.3% (*n* = 130/144) of respondents reporting that they rarely or never performed these techniques in birds.

### 3.3. Local Anaesthetics and Equipment

There were significant differences in the use of local anaesthetics between ACZM/ECZM and ACVAA/ECVAA specialists/specialists-in-training. Lidocaine was used more frequently by ACZM/ECZM specialists/specialists-in-training (65.1%, *n* = 28/43 vs. 32.9%, *n* = 50/152; *p* < 0.001), whereas a higher proportion of ACVAA/ECVAA specialists/specialists-in-training reported using ropivacaine (21.7%, *n* = 33/152 vs. 2.4%, *n* = 1/42) and bupivacaine/levobupivacaine (54.6%, *n* = 83/152 vs. 16.3%, *n* = 7/43; *p* < 0.001). Additionally, a higher proportion of ACZM/ECZM specialists/specialists-in-training reported that they used mixtures of local anaesthetics (23.8%, *n* = 10/42 vs. 4.0%, *n* = 6/152; *p* < 0.001).

Regarding the equipment that was used to perform locoregional anaesthesia, a higher proportion of ACVAA/ECVAA specialists/specialists-in-training used Tuohy (21.7%, *n* = 33/152 vs. 9.5%, *n* = 4/42; *p* < 0.001) and spinal needles (42.8%, *n* = 65/152 vs. 16.7%, *n* = 7/42; *p* < 0.001), as well as ultrasonography (30.9%, *n* = 47/152 vs. 4.8%, 2/42; *p* = 0.009) and electrical nerve stimulators (40.1%, *n* = 61/152 vs. 7.1%, *n* = 3/42; *p* < 0.001) when performing locoregional anaesthesia on the animals under their care. A higher proportion of ACZM/ECZM specialists/specialists-in-training used hypodermic needles for neuraxial and peripheral nerve blocks (61.9%, *n* = 26/42 vs. 25.0%, *n* = 38/152; *p* < 0.001).

### 3.4. Future Developments and Identification of Useful Research Areas

It was found that 68.1% (*n* = 284/417) of respondents reported that they were somewhat hesitant to perform locoregional anaesthesia in non-conventional species on a routine basis. Common reasons for this were a lack of confidence in performing a specific technique (80.3%, *n* = 228/284), limited information on both the clinical effects of the blocks and appropriate doses of local anaesthetics (76.4%, *n* = 217/284), concerns for possible complications and unwanted effects (64.8%; *n* = 184/284) and lack of availability of specialised equipment such as ultrasound machines or electrical nerve stimulators (50.4%; *n* = 143/284).

The most frequently cited area of research or future development was dental block techniques for rabbits and rodents, with 81.1%, *n* = 338/417 and 71.0%, *n* = 296/417 of respondents identifying these techniques as being either very important or extremely important. Locoregional anaesthetic techniques involving the wings of birds were also identified as an important area of future development, with 66.2% (*n* = 267/417) of respondents rating this area of research as either very important or extremely important. The importance of further research into the use of dental blocks in reptiles was perceived differently between the two groups of specialists, with a higher proportion of ACVAA/ECVAA specialists/specialists-in-training (21.9%, *n* = 41/187 vs. 9.8%, *n* = 5/51; *p* = 0.021) rating this area of research as being either very important or extremely important.

The proportion of participants who considered ultrasonography absolutely necessary when performing locoregional anaesthesia in non-conventional species was higher among ACVAA/ECVAA specialists/specialists-in-training compared to ACZM/ECZM specialists/specialists-in-training (77.0%, *n* = 144/187 vs. 49.0%, *n* = 23/51; *p* = 0.001).

## 4. Discussion

The main findings of this study were that locoregional anaesthesia is still relatively underused in non-conventional pet species compared to domestic species, particularly in reptiles and birds. Possible reasons for this are the lack of information around species-specific locoregional techniques, and the paucity of scientific evidence with respect to the safe and effective use of local anaesthetics in many non-domestic species. This study also documented the different attitudes of board-certified specialists in anaesthesiology and zoological medicine towards the use of local anaesthetics and equipment, as well as differing perceptions of which areas need further development and research.

With respect to the reportedly low use of locoregional techniques in reptiles and birds compared to non-conventional mammalian species, this might reflect the higher frequency with which the latter group are presented to veterinary practices and anaesthetised to facilitate various procedures, rather than a reluctance of veterinarians to perform locoregional blocks specifically in reptiles and birds. Nevertheless, the vast anatomical and physiological differences between different reptile orders, suborders, families and species make it extraordinarily challenging to obtain a body of literature sufficient to cover all the animals included in this taxonomic order. Interestingly, the use of epidural/spinal anaesthesia was uncommon in rabbits, despite this species being a model for research on neuraxial techniques [19,20].

There were differences in the preferences of local anaesthetic agents and their mixtures between veterinarians with different areas of expertise. Interestingly, the specialists/specialists-in-training in zoological medicine preferred lidocaine over longer-lasting anaesthetic agents such as ropivacaine and bupivacaine/levobupivacaine, which were used more often by anaesthesiologists. Although this study did not explicitly ask about the reasons for someone’s drug preference, a possible explanation for this finding may be the anaesthesiologists’ greater familiarity with the use of local anaesthetic agents which have longer durations of effect but are generally considered to be “less safe” than lidocaine based on reported toxic doses in other species [21]. In these authors’ opinion, other explanations may be related to cost, with lidocaine being less expensive than some longer-acting local anaesthetic alternatives, or anaesthesiologists tend to be involved in performing anaesthesia for longer procedures compared to specialists/specialists-in-training in zoological medicine, and they may prefer to use longer-lasting agents for this reason.

ACZM/ECZM specialists/specialists-in-training reportedly mixed different local anaesthetic agents together prior to use more frequently than anaesthesists did. One possible reason for this may be a desire to combine a quicker onset of action with a prolonged duration of effect by combining short- and long-acting drugs [22]; however, there is no strong evidence to support this approach [23].

In relation to locoregional anaesthesia equipment, the greater popularity of ultrasonography among anaesthesiologists compared to the AZCM/ECZM specialists/specialists-in-training group may be explained by the dramatic increase in the use of ultrasound-guided locoregional anaesthesia in small animals over the past ten years, during which time most veterinary anaesthesiologists have become very familiar with the use of ultrasonography to facilitate a wide range of nerve blocks in their patients [24,25,26]. Moreover, anaesthesiologists tended to use specialised supplies such as Tuohy needles and spinal needles to perform both neuraxial and peripheral nerve techniques, whereas most zoological medicine specialists preferred to use hypodermic needles. Possible explanations for this finding could be overall greater familiarity with their use and/or anaesthesiologists having easier access to locoregional anaesthesia-specific equipment as a result of them already being used in small animals [27,28].

This study has some limitations, the most important of which is that the sample population may not be fully representative of the whole. Only veterinarians with access to internet could participate in the survey, which, considering that internet technology has diffused unevenly across countries, could represent a selection bias by excluding veterinarians from developing countries. Despite our efforts to advertise the survey to this category of specialists, the zoological medicine specialists/specialists-in-training were underrepresented compared to anaesthesiologists, which could have potentially affected the validity of some study findings. Moreover, when comparing groups of different specialists, only defined categories—namely, the ACVAA/ECVAA and ACZM/ECZM diploma holders and specialists-in-training—could be included, which resulted in the exclusion of a considerable number of responses from part of the data analysis. Unfortunately, this loss of data was unavoidable as one fundamental assumption of the Chi-squared test of association (which was used in this study to investigate differences between proportions) is that all data must be categorised into mutually exclusive categories, with no overlap [29,30]. The inclusion in this analysis of veterinarians not grouped in specific categories, and potentially involved in both anaesthesia and exotic animals general medicine, would have broken that assumption and therefore jeopardised the validity of our results.

Another potential limitation of this study is that the overall response rate could not be calculated. According to the CHERRIES guidelines, a closed-survey design enables the researchers to quantify the target population and precisely calculate the response rate. In order to accomplish this, the participants should have accessed the survey solely via a personalised email-invitation and advertisement of the survey to open groups of practitioners through social media should have been avoided. Nevertheless, the drawbacks of this approach would have been the inability to reach the large number of specialists whose contact details are unavailable, as well as excluding many non-board-certified veterinarians that may practice anaesthesia or/and exotic animals medicine at advanced level. The responses from this unspecified category of veterinarians were deemed valuable by these authors and were included in the general data analysis. Authors should discuss the results and how they can be interpreted from the perspective of previous studies and of the working hypotheses. The findings and their implications should be discussed in the broadest context possible. Future research directions may also be highlighted.

## 5. Conclusions

Locoregional anaesthesia continues to be underused in non-conventional animal species; however, its use is more common in rodents, rabbits and ferrets compared to reptiles and birds. As has been carried out in many domestic species over the past ten years, prospective studies focusing on species-specific locoregional anaesthetic techniques and the clinical pharmacology of local anaesthetics are needed to increase the spread of locoregional anaesthesia to non-conventional animal species. To this end, specific blocks of which the development was perceived as particularly important to improve the care of non-conventional animals are dental blocks for rabbits and rodents and blocks for the wings of birds.

## Data Availability

Raw data may be provided by the corresponding author upon reasonable request.
